# The Influence of in Vitro Gastrointestinal Digestion of *Brassica oleracea* Florets on the Antioxidant Activity and Chlorophyll, Carotenoid and Phenolic Content

**DOI:** 10.3390/antiox8070212

**Published:** 2019-07-10

**Authors:** Teodora Scrob, Anamaria Hosu, Claudia Cimpoiu

**Affiliations:** Research Center for Advanced Chemical Analysis, Instrumentation and Chemometrics (ANALYTICA), Department of Chemistry, Faculty of Chemistry and Chemical Engineering, Babeş-Bolyai University, 11 Arany Janos Street, 400028 Cluj-Napoca, Romania

**Keywords:** *Brassica oleracea*, ultrasound-assisted extraction, kinetic model, in vitro digestion, antioxidant activity, chlorophylls, carotenoids, total phenolic content

## Abstract

*Brassica oleracea* L. var*. Italica* is known to contain a wide variety of antioxidants and due to the protection against various diseases its consumption has been increasing over the years. Thus, knowledge of the changes that occur during the digestion process is of great interest. The aim of this study was to investigate the influence of in vitro gastrointestinal digestion of broccoli on antioxidant activity and on the chlorophyll, carotenoid and phenolic content. First, the ultrasound-assisted extraction of bioactive compounds was optimized and the kinetic model was evaluated. Then, the broccoli was subjected to a static simulated digestion. The antioxidant activity was monitored by ABTS [2,2’-azinobis-(3-ethylbenzothiazoline-6-sulfonate)] assay and the contents of target compounds were investigated by UV-Vis spectrophotometry and thin-layer chromatography. The optimum conditions were: solvent—ethanol; time—20 min and temperature—30 °C, and a second order kinetic model was found to describe the mechanism of extraction. The antioxidant activity and carotenoid, chlorophyll and total phenolic content was significantly decreased after simulated gastric and intestinal digestion. The gastric digestion considerably decreased carotenoid and chlorophyll content, meanwhile the intestinal digestion significantly decreased the total phenolic content (TPC). The antioxidant activity was equally affected by both gastric and intestinal digestion.

## 1. Introduction

Broccoli (*Brassica oleracea)* is known to contain a wide variety of antioxidants which may provide protection against the development of a number of diseases [1]. Broccoli (*Brassica oleracea* L. var. *Italica*) consumption has been increasing over the years due to its health-promoting compounds. Therefore there is a growing interest in the knowledge of the physicochemical and biochemical changes that occur in this vegetable during the digestion process. Broccoli heads are rich in minerals and vitamins, and fiber and health related secondary metabolites [1]. Consumption of cruciferous vegetables such as broccoli has been shown to reduce the risk of several types of cancer and cardiovascular disease mortality [1,2]. One important group of bioactive compounds found in broccoli are carotenoids. They are of great importance for both their nutritional and physiological activities and also because they cannot be synthesized by animals. Consequently they should be acquired through the diet [3]. However, is difficult to consider broccoli as an important source of carotene because it contains little fat, which is necessary for the absorption of carotene. Chlorophylls are some other important compounds that have been studied in broccoli heads, florets and sprouts [4]. Chlorophylls (Chl-A and Chl-B) are photosynthetic pigments that are widely distributed in nature being important components of the photosynthetic membranes. Also, polyphenols, both low molecular weight with single aromatic-ringed compounds and complex tannins and derived polyphenols, can be found in broccoli. They can be found in a free state or commonly conjugated to sugars and organic acids. The main activity reported for polyphenols has been as antioxidants, although they also have anti-inflammatory, anti-carcinogenic, anti-ageing and anti-thrombotic effects [1]. 

Not all of the bioactive compounds present in food are eventually to be absorbed by the human body because of the different physicochemical and biochemical conditions during the digestion process [5]. Knowledge of the physicochemical changes that occur in food during digestion would be very helpful for not only the food industry. Because human nutritional studies are expensive, long lasting and also ethically restricted, in vitro methods simulating digestion processes have been recently developed. Simulated digestion methods typically include the oral, gastric and small intestinal phases, and occasionally large intestinal fermentation. These methods try to mimic physiological conditions in vivo, taking into account the presence of digestive enzymes and their concentrations, pH, digestion time, and salt concentrations [6].

To achieve their effects, bioactive compounds present in different types of food must be released from the food matrix in the gastrointestinal tract and must be bioavailable [7]. Bioavailability describes the fraction of a compound that is released, absorbed and reaches the systemic circulation [8]. Bioavailability is a key step regarding foods and the benefits of their components that leads to understanding of the mechanisms of action in relation to the benefit [9]. Digestion starts in the mouth, where mastication and the action of salivary enzymes take place.It continues in the stomach and small intestine, where nutrients and bioactive compounds become available for absorption through the intestinal wall. All these physiological conditions may induce structural changes that can affect the bioavailability of food constituents. During gastrointestinal digestion (GI), some bioactive compounds can be further degraded or metabolized and these structural changes could affect their bioactivity [10]. Due to the complexity of food compounds, the many factors affecting their transition during digestion and the different mechanisms of absorption of molecules, unraveling the bioavailability of food constituents is challenging [9]. Also, to understand the relationship between food and nutrition it is important to evaluate the bioavailability of various components of food.

Even if *Brassica oleracea* is considered as a potential source of antioxidants and bioactive compounds, few studies have reported on the impact of digestion on these compounds found specifically in broccoli. Such studies are important because only those compounds released from the solid matrix as a consequence of digestion are potentially bioavailable [5]. For these reasons, the investigation of in vitro GI digestion on the stability and recovery of chlorophylls, carotenoids and total polyphenols from *Brassica oleracea* and the changes in the amount of bioactive compounds and in the AA before and after in vitro digestion were very important to be performed.

Thus, the aim of this study was to investigate the effect of in vitro GI digestion on the stability and recovery of chlorophylls, carotenoids and total polyphenols in *Brassica oleracea.* Simulated digestion was categorized into salivary, gastric and intestinal, using a standardized static in vitro digestion method. The changes in the number of bioactive compounds and in the total antioxidant activity (AA) before and after in vitro digestion were also evaluated. In the first phase of our research, the optimization of chlorophyll and carotenoid extraction from *Brassica oleracea* and evaluation of the kinetic model that could be applied in order to assess optimum performance of the extraction process was performed. These data may contribute to the promotion of this vegetable in the food industry and to reassure its consumers about its beneficial health effects.

## 2. Materials and Methods 

### 2.1. Chemicals

Ethanol, acetone, 2,2′-Azino-bis(3-ethylbenzothiazoline-6-sulfonic acid) diammonium salt (ABTS), Folin–Ciocalteu reagent, KCl, NaHCO_3_, KH_2_PO_4_, (NH4)_2_CO_3_, NaCl, HCl, MgCl_2_·(H_2_O)_6_ and thin-layer chromatography (TLC) plates were purchased from Merck (Darmstadt, Germany). α-amylase, pepsin from porcine gastric mucosa, pancreatin from porcine pancreas and bile salts were purchased from Alfa Aesar (Karslruhe, Germany). All reagents used in the experimental part were of analytical purity.

### 2.2. Plant Material

Broccoli (*B. oleracea*, var. *italica*) florets were purchased from a local supermarket in Cluj-Napoca, Romania. Prior to extraction of chlorophylls and carotenoids, broccoli florets were cut into small pieces and ground in order to increase the extraction yield. To avoid pigment degradation, chlorophylls and carotenoids were analyzed as soon as they were extracted. The extraction of target compounds was achieved in the ultrasonic thermostatic bath Elmasonic E60H (Elma Schmidbauer GmbH, Singen am Hohentwiel, Germany). The extracts were centrifuged using a Centurion Scientific centrifuge C2006 (Centurion Scientific Limited, Bosham, UK).

### 2.3. Ultrasonic Assisted Extraction (UAE) and Kinetic Modeling

The extraction was performed on 0.5 g accurately weighted broccoli that was mixed with 5 mL solvent (ethanol or acetone) into a centrifuge flask. The samples were incubated in the ultrasonic thermostatic bath Elmasonic E60H (Elma Schmidbauer GmbH, Singen am Hohentwiel, Germany)at three constant temperatures (30 °C, 50 °C and 80 °C) for 5, 10, 15, 20, 30 and 40 min. Then, the extracts were centrifuged at 875 g for 10 min and the supernatants were collected and used for further analyses.

In order to describe the mechanism of ultrasound extraction, a second order kinetic model was investigated. Because solid–liquid extraction can be considered as the reverse of the adsorption process, a second-order rate law could be applied to the UAE process [11]:

(1) $$\frac{\mathrm{dCt}}{\mathrm{dt}}=k\times {\left(\mathrm{Ce}-\mathrm{Ct}\right)}^{2}$$

where *k* is the second order extraction rate constant (L/g·min), *C_e_* is the equilibrium concentration of broccoli pigments in the liquid extract (mg/L) (extraction capacity) and *C_t_* is broccoli pigments (mg/L) in the liquid extract at a given extraction time *t*.

The second order law for UAE process can be integrated, obtaining the following equations:

(2) $$\mathrm{Ct}=\frac{{k\times t\times C}_{e}^{2}}{1+k\times t\times \mathrm{Ce}}$$

(3) $$\frac{t}{\mathrm{Ct}}=\frac{1}{{k\times C}_{e}^{2}}+\frac{t}{\mathrm{Ce}}=\frac{1}{h}+\frac{t}{\mathrm{Ce}}$$

where *h* is the initial extraction rate (g/L·min) when *t* approaches 0.

### 2.4. Spectrophotometric Measurements

Spectrophotometric measurements were performed at room temperature, in triplicate using a T80+ UV-Vis spectrophotometer (PG Instruments, Lutterworth, UK).

#### 2.4.1. Quantification of Chlorophylls and Carotenoids

Chlorophyll and carotenoid determination was carried out according to the spectrophotometric methods described by Lichtenthaler and Buschmann [12]. The absorbance of samples was measured at 470, 648 and 664 nm. Content of chlorophylls (Chl-A and Chl-B) and carotenoids (mg/mL) found in broccoli was calculated using the following equations [12]:

Chl-A = 13.36A_664_ − 5.19A_648_ (4)

Chl-B = 27.43A_648_ − 8.12A_664_ (5)

Carotenoids total = (1000A_470_ − 1.63Chl-A - 104.96Chl-B) / 221 (6)

where *A* is the absorbance.

For further studies the following conditions were chosen for extraction: solvent—ethanol, temperature—30 °C and time—20 min. 

#### 2.4.2. Total Phenolic Content

Total phenolic content (TPC) was determined according to the Folin–Ciocalteu assay with slight modification [13]. First, 0.3 mL extract diluted five times was mixed with 1.5 mL Folin–Ciocalteu’s reagent (0.2 N). After 5 min, 1.2 mL sodium carbonate (0.7 M) was added and the mixture was incubated for 2 h in dark conditions at room temperature. The absorbance was measured at 760 nm and the TPC was determined from the calibration curve of gallic acid being expressed as mg gallic acid/mL of extract.

#### 2.4.3. Antioxidant Activity Determination

Antioxidant activity (AA) of broccoli samples was determined according to ABTS assay with some modifications [14]. The ABTS•^+^ solution was freshly diluted with distilled water till it had an absorbance of 0.8–0.9 at 734 nm. Then 0.5 mL of five times diluted extract was mixed with 3 mL ABTS•^+^ solution. The mixtures were incubated in dark conditions at room temperature for 15 min, after which the absorbance of sample was read at 734 nm. The AA was calculated on the basis of calibration curve and expressed as Trolox equivalents (μmols/mL).

### 2.5. TLC Analysis

Thin-layer chromatography (TLC) was carried out using the CAMAG (Basel, Switzerland) devices: twin-through chamber, Linomat 5 applicator, TLC visualizer-Digistore 2. Chromatographic separation was performed on a silica gel 60F_254_ HPTLC plate. Aliquots of 10 µL of each sample were applied as 8mm bands, at 1.5 cm above the low edge of the plate, using a 100 nL/s flow rate. The plates were developed in pre-saturated twin-trough chamber using a mixture of benzene-ethyl acetate-methanol 70:25:5, *v/v/v* as the mobile phase. The compounds were detected in white light and in UV light at 366 nm. 

### 2.6. In Vitro Simulated GI Digestion

A static model that simulates GI digestion was performed according to protocol developed by Minekus et al. [6]. The samples were independently treated for sequentially simulating the mouth, stomach and small intestine digestion. The detailed composition of simulated salivary fluid—SSF, gastric fluid—SGF and intestinal fluid—SIF is given in Table 1.

**Figure 1:**
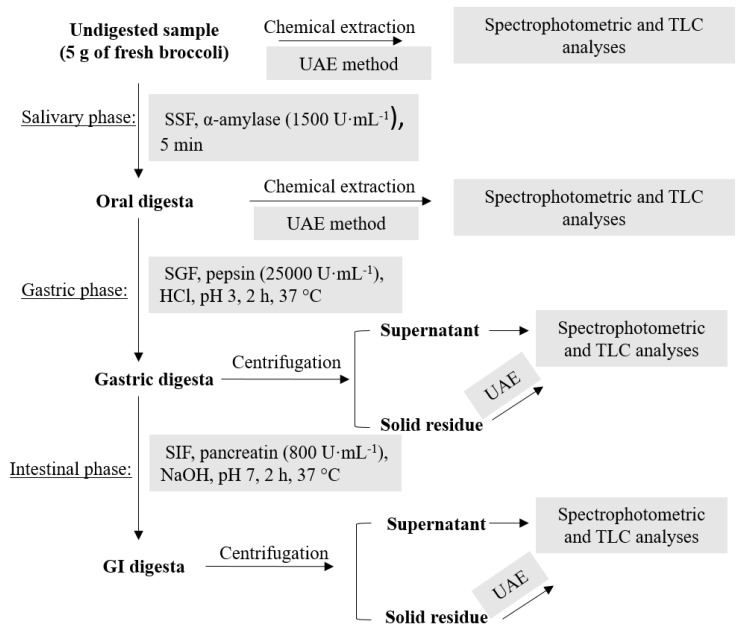
Graphic representation of simulated gastrointestinal digestion (GI) and further analyses. TLC: thin-layer chromatography; UAE: ultrasonic assisted extraction; SSF: simulated salivary fluid; SGF: simulated gastric fluid; SIF: simulated intestinal fluid.

Graphical representation of the in vitro gastrointestinal digestion model carried out on broccoli samples is shown in Figure 1.

The oral digestion was performed on 5 g of raw broccoli that was mixed with 3.5 mL of SSF in order to form a paste of a thin consistency in which the ratio of food to SSF was 50:50 (w/v). Then, 0.5 mL of α-amylase solution (1500 U·mL^−1^ in SSF), 25 µL of CaCl_2_ 0.3 M and 975 µL of distilled water was added. For the gastric digestion, 7.5 mL of SGF, 1.6 mL of pepsin solution (25,000 U·mL^−1^ in SGF), 5 µL of CaCl_2_ 0.3 M and 0.695 µL of distilled water was added to the oral bolus. The pH was reduced to 3.0 by adding 1 M HCl solution (0.2 mL). The mixture was mixed on a magnetic stirrer for 2 h at 37 °C. In the case of the intestinal phase, the gastric chyme was mixed with 11 mL SIF, 5.0 mL of pancreatin solution (800 U·mL^−1^ in SIF), 2.5 mL of 160 mM fresh bile, 40 µL of 0. 3M CaCl_2_, 0.15 mL of 1 M NaOH and 1.31 mL of water. The NaOH was added in order to neutralize the mixture to pH 7.0. The digestion mixture was stirred again for 2 h at 37 °C. After both gastric and intestinal digestion, the samples were centrifuged 10 min at 875 g and the supernatants were collected for further analysis. The solid residues were extracted as original samples in order to check for the remaining compounds. In the case of the salivary phase mixture, the extraction of remaining compounds was performed directly on the sample without centrifugation due to the high viscosity of mixture.

### 2.7. Statistics

The results are presented as mean ± standard deviations of three replicates. The results were statistically processed using one-way variance analysis (ANOVA) by means of StatistiXL 2.0 (Digital River, Broadway-Nedland, Australia) and the differences were considered statistically significant if *p* < 0.05 for probability P ≥ 95.

## 3. Results and Discussion

### 3.1. UAE of Chlorophylls and Carotenoids from Brassica Oleracea

#### 3.1.1. Optimization of UAE

**Figure 2:**
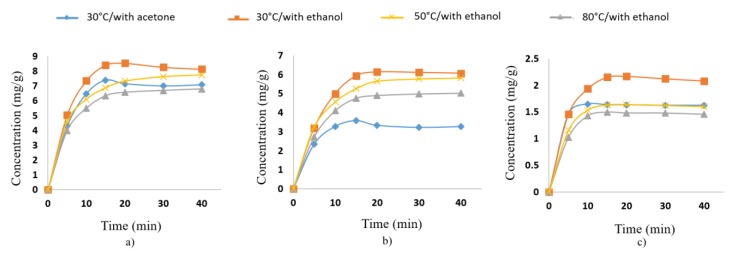
Influence of extraction time, solvent and temperature on UAE of Chl-A (**a**), Chl-B (**b**) and carotenoids (**c**) from *Brassica oleracea*.

The influence of extraction time, temperature and solvent on the UAE of chlorophylls and carotenoids from *Brassica oleracea* was studied in order to optimize the conditions of extraction. Acetone and ethanol were chosen as extraction solvents because these solvents were often used for the extraction of food pigments [15]. Ethanol revealed a better efficiency for chlorophyll (Figure 2a,b) and carotenoid extraction (Figure 2c). Consequently, ethanol was chosen as the solvent for extraction of target compounds from broccoli and from samples obtained after the simulated digestion experiment. Ethanol was also preferred because of its recommendation as an eco-friendly and safe solvent and the possible applications of the extracts in food or pharmaceutical industries [16].

The tested extraction time chosen was between 5 and 40 min because some studies report that the effect of UAE is more effective in the first 30 min [17]. For all compounds, the UAE yield was significantly time dependent and increased with extended ultrasonic times, especially from 5 to 20 min, but more slowly from 20 to 40 min. Figure 2 shows the change in extraction yield of broccoli pigments at different extraction times using ethanol as the extraction solvent. As it can be seen, the most efficient extraction period for obtaining maximum content of chlorophylls and carotenoids from broccoli was about 20 min. This fact was also observed by other authors [18] reporting that with an increase in the extraction time, the solvent becomes saturated with the product and after that there is negligible mass transfer and extraction.

The effect of temperature on extraction yield was studied by performing the extraction at 30 °C, 50 °C and 80 °C. The best extraction yield was obtained at 30 °C, as can be seen in Figure 2. It was observed that increasing temperature above 30 °C led to a decreasing trend of the extraction yield. This is due to the two phenomena that play an important role in UAE, namely cavitation effect and thermal effect. At 30 °C, both the effects were equally dominating and the combination effect resulted into relatively higher extraction yield.

#### 3.1.2. Kinetic Modeling

**Figure 3:**
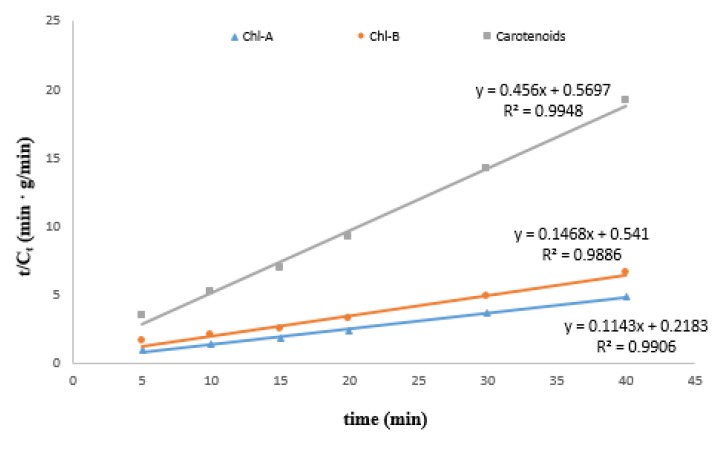
Validation of the second order kinetic model.

The experimental data from the UAE of Chl-A, Chl-B and carotenoids from broccoli at 30 °C using ethanol were processed and plotted in the specific coordinates of the second-order kinetic model (Figure 3).

The specific kinetic parameters, such as extraction capacity (concentration at saturation—*C_e_*), extraction rate constant (*k*) and initial extraction rate (*h*) were determined (Table 2). Fitting of the second-order kinetic model for all experimental data was confirmed by the value of determination coefficient (R^2^ > 0.98). The second-order kinetic model was also used to describe the release kinetics of other bioactive compounds. For example, Qu et al. [19] also used the second-order model to describe the solid–liquid extraction processes of antioxidants from pomegranate marc. Using this model, it was observed that temperature has the most predominant influence on the *C_s_*, *h*, *k* predicted parameters. Thus, the second-order kinetic model can be used to describe the extraction processes under different operating conditions and parameters of UAE.

### 3.2. In Vitro Simulated GI Digestion

The effects of bioactive compounds depend not only on their concentration in raw vegetables but they depend on what happens with these compounds after digestion. Using fresh broccoli, there was an interest in finding out if ethanolic extractions underestimate or overestimate chlorophylls and carotenoids, total available polyphenol concentrations and AA compared to in vitro GI digestion. An in vitro GI digestion procedure was performed to investigate the changes in AA, TPC and chlorophylls and carotenoids present in *Brassica oleracea*.

The results obtained after in vitro GI digestion of broccoli are shown in Table 3. All the results are calculated taking into account the dilution factor of the sample in order to make a good comparison and investigation.

The levels of target compounds (Table 3) were analyzed in ethanolic extracts of broccoli and in their digesta of salivary, gastric and intestinal phases. The salivary digesta ensure food fragmentation and impregnation with saliva in order to obtain the semi-solid bolus. After extraction of compounds from bowl any statistically significant changes in the content of target compounds were observed. As far as the carotenoids are concerned, it was found that the content was aproximatively the same in both gastric and intestinal phase. Compared to content before digestion (6.11 mg/mL), there was a loss of 78.07% after the whole digestion. There are studies that report carotenoids are more sensitive to acidic conditions than to alkaline conditions [20]. This aspect was also observed for carotenoids present in broccoli with regard to their instability at low pH values (gastric conditions).However, the in vitro bioaccessibility varies widely for different carotenoids in a given food as well as for a given carotenoid in different foods [20]. Even if in vitro models are increasingly used and may be relevant in the food industry, exceptions do exist and in vitro data are not fully concordant with in vivo responses [21]. Changes in carotenoid content is not significant during the in vitro intestinal digestion as compared to gastric digestion (*p* > 0.05).

Experimental data also support the general outline of chlorophyll’s digestive behavior (Table 3). Concerning the chlorophylls, it was found that the highest chlorophyll content after the in vitro digestion process was found in the gastric phase and the lowest content was in the intestinal phase. The sensitivity of chlorophyll to acidic conditions can be observed here too, the gastric environment playing an important role for significant chlorophyll modification. Studies reported that gastric acidity determined chlorophyll’s transformation into Mg^2+^-free derivates (including pheophytins and pyropheophytins) [22] or chlorophylls were subjected to an oxidized reaction during digestion [23]. However, compared to the content before digestion (18.21 mg/mL), a higher loss after the whole digestion (72.05%) was observed. This fact could suggest that chlorophylls are sensitive under in vitro conditions.

**Figure 4:**
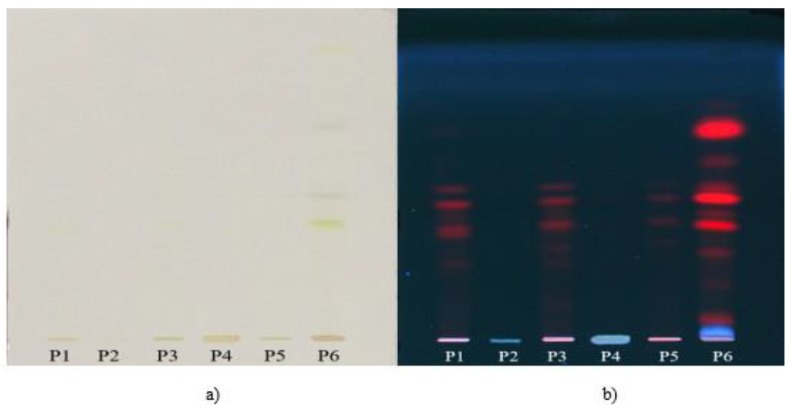
The image of TLC separation in visible light (**a**) and in UV light at 366 nm (**b**) of salivary phase digesta (P1), gastric phase digesta (P2), gastric ethanolic extract (P3), intestinal phase digesta (P4), intestinal ethanolic extract (P5) and non-digested ethanolic extract (P6).

The changes occurring in the content of chlorophylls and carotenoids before and after each step of in vitro digestion was also evaluated by TLC. The extracts—salivary phase digesta (P1), gastric phase digesta (P2), gastric ethanolic extract (P3), intestinal phase digesta (P4), intestinal ethanolic extract (P5) and non-digested ethanolic extract (P6)—were analyzed by TLC and a decreasing trend in chlorophyll and carotenoid content after in vitro digestion process was observed. The chromatograms were visualized in visible light (Figure 4a) and UV light at 366 nm (Figure 4b).

White light (Figure 4a) confirmed the presence of chlorophylls and carotenoids in the non-digested ethanolic extract (P6), but it could not predict the following steps of digestion due to the low sensibility of this detection. The chromatogram visualized at 366 nm (Figure 4b) also confirmed the presence of compounds of interest in the non-digested sample (P6). However, the absence of these compounds both in the gastric (P2) and intestinal phase (P4) (Figure 4b) suggests a possible degradation or transformation of these compounds into other derivates during the in vitro digestion process. On the image of the chromatogram at 366 nm (Figure 4b) there could also be observed a decreasing trend of chlorophylls and carotenoids in the ethanolic extracts of the solid digested residues (P1, P3, P5), which suggests the majority of the compounds have been released from the broccoli matrix during digestion and subjected to digestion process changes.

Regarding TPC it is well known that polyphenols are highly metabolized during digestion, for example oxidized or hydrolyzated, and transformed into metabolites completely different from the initial compounds. Also, polyphenols can interact with different dietary constituents such as proteins, fibers and lipids, changing their chemical structure and affecting in this way their bioavailability [24]. In the present study, the release of polyphenols from *Brassica oleracea* following the simulated digestion was mainly achieved during the intestinal phase. While chlorophylls and carotenoids showed a large decrease in the gastric phase, polyphenols are more stable in gastric conditions, but their content decreases more in the intestinal phase (45.20%).

The AA of all the samples was lower after in vitro digestion as compared to the non-digested samples following the same trend. The low AA at the end of the simulated digestion process could indicate a low stability of compounds to pH change and enzymatic activity. The lower AA obtained during digestion might be due the biotransformation of compounds to others with low antioxidant potential [25] or by formation of new antioxidant metabolites.

## 4. Conclusions

After a simulated in vitro GI digestion both broccoli pigments, polyphenols and antioxidant activity seem to be affected during digestion process. The results indicate that the simulated digestion decreased total carotenoid and chlorophyll content from *Brassica oleracea*, as well as total polyphenol content and antioxidant activity. All changes are statistically significant (*p* < 0.05), with the exception of carotenoids modifying during intestinal digestion. The obtained results represent a basis for further studies on the stabilization of bioactive compounds, their potential health benefits and the use of broccoli as a potential functional ingredient in the food industry. Moreover, the results support the opinion that such studies could be an alternative to the use of animals and humans for food digestibility and bioavailability screening.

## Figures and Tables

**Table 1:** Preparation of simulated digestion fluids. SSF: salivary fluid; SGF: gastric fluid; SIF: intestinal fluid.

	Volume of Constituents (mL)					
Simulated Digestion Fluid	KCl (37.3 g/L)	KH_2_PO_4_ (68 g/L)	NaHCO_3_ (84 g/L)	NaCl (117 g/L)	MgCl_2_(H_2_O)_6_ (30.5 g/L)	(NH_4_)_2_CO_3_ (48 g/L)
SSF (pH = 7)	15.1	3.7	6.8	__	0.5	0.06
SGF (pH = 3)	6.9	0.9	12.5	11.8	0.4	0.5
SIF (pH = 7)	6.8	0.8	42.5	9.6	1.1	__

**Table 2:** Kinetic parameters for the second-order kinetic model.

Compound	B = 1/C_e_	A = 1/h	C_e_ = 1/B (mg/g)	h = 1/A	k = h/(C_e_)^2^ (g/mg·min)	R^2^
Chl-A	0.1143	0.2183	8.7490	4.5809	0.0598	0.9906
Chl-B	0.1468	0.5410	6.8120	1.8484	0.0398	0.9886
Carotenoids	0.4560	0.5697	2.1930	1.7553	0.3650	0.9948

**Table 3:** Total content of carotenoids, chlorophylls and phenolics, and antioxidant activity in extract of raw broccoli and sample subjected to simulated GI digestion.

Digestion Phase	Concentration			Antioxidant Activity (µmol/mL)
	Carotenoids (mg/mL)	Chlorophylls (mg/mL)	Polyphenols (µg/mL)	
**Initial**	6.11 ± 0.98	18.21 ± 1.21	136.44 ± 9.85	1.05 ± 0.03
Gastric	1.34 ± 0.12	7.49 ± 0.84	126.36 ± 5.16	0.43 ± 0.01
Intestinal	1.34 ± 0.14	5.09 ± 0.62	69.24 ± 4.25	0.20 ± 0.00
